# Peripheral Blood Mononuclear Cells Mitochondrial Respiration and Superoxide Anion after Heart Transplantation

**DOI:** 10.3390/jcm11237247

**Published:** 2022-12-06

**Authors:** Abrar Alfatni, Anne-Laure Charles, François Sauer, Marianne Riou, Fabienne Goupilleau, Samy Talha, Alain Meyer, Emmanuel Andres, Michel Kindo, Jean-Philippe Mazzucotelli, Eric Epailly, Bernard Geny

**Affiliations:** 1 Team 3072 “Mitochondria, Oxidative Stress and Muscle Protection”, Translational Medicine Federation of Strasbourg (FMTS), Faculty of Medicine, University of Strasbourg, 11 Rue Humann, 67000 Strasbourg, France; 2 Physiology and Functional Exploration Service, University Hospital of Strasbourg, NHC, 1 Place de l’Hôpital, CEDEX, 67091 Strasbourg, France; 3 Department of Internal Medicine, University Hospital of Strasbourg, 1 Place de l’Hôpital, CEDEX, 67091 Strasbourg, France; 4 Cardiovascular Service, University Hospital of Strasbourg, NHC, 1 Place de l’Hôpital, CEDEX, 67091 Strasbourg, France

**Keywords:** PBMCs, mitochondrial respiration, heart transplantation, oxidative stress, cardiac function

## Abstract

Introduction: The mitochondrial function of circulating peripheral blood mononuclear cells (PBMCs) is an interesting new approach to cardiac diseases. Thus, PBMC’s mitochondrial respiration decreases in relation to heart failure severity. However, no data are available on heart-transplanted patients (Htx). Population and Methods: We determined PBMCs mitochondrial respiration by high-resolution respirometry (Oroboros Instruments) and superoxide anion production using electron paramagnetic resonance (Bruker-Biospin) in 20 healthy subjects and 20 matched Htx and investigated clinical, biological, echocardiographic, coronarography and biopsy characteristics. Results: PBMCs mitochondrial respiratory chain complex II respiration was decreased in Htx (4.69 ± 0.84 vs. 7.69 ± 1.00 pmol/s/million cell in controls and Htx patients, respectively; *p* = 0.007) and complex IV respiration was increased (24.58 ± 2.57 vs. 15.68 ± 1.67 pmol/s/million cell; *p* = 0.0035). Superoxide anion production was also increased in Htx (1.47 ± 0.10 vs. 1.15 ± 0.10 µmol/min; *p* = 0.041). The leucocyte-to-lymphocyte ratio was increased in Htx, whom complex II correlated with leucocyte number (r = 0.51, *p* = 0.02) and with the left ventricular posterior wall peak early diastolic myocardial velocity (r = −0.62, *p* = 0.005). Complex IV was increased in the two patients with acute rejection and correlated negatively with Htx’s isovolumetric relation time (r = −0.45, *p* = 0.045). Discussion: Although presenting with normal systolic function, Htx demonstrated abnormal PBMC’s mitochondrial respiration. Unlike immunosuppressive therapies, subclinical diastolic dysfunction might be involved in these changes. Additionally, lymphopenia might reduce complex II, and acute rejection enhances complex IV respirations. Conclusion: PBMC’s mitochondrial respiration appears modified in Htx, potentially linked to cellular shift, mild diastolic dysfunction and/or acute rejection.

## 1. Introduction

Despite the significant progress made in recent years in the treatment of patients presenting with preserved--or reduced--ejection fraction, many of them still reached terminal heart failure characterized by a poor short-term prognosis [1]. In this context, heart transplantation remains the treatment of choice, allowing major increases in life quality and duration. Nevertheless, a better knowledge of heart transplant pathophysiology appears interesting in order to improve cardiac function and reduce rejection of the graft, which remains a significant issue. The usual clinical, biological and echocardiographic follow-up does not always allow an early diagnosis, urging, therefore, to perform a regular cardiac biopsy and coronary artery angiography [2].

Recent data demonstrated that myocardial mitochondrial function was impaired in Htx presenting with cardiac allograft vasculopathy, supporting interest in mitochondrial functions analysis after heart transplantation [3]. The mitochondrion is the main producer of cell energy, coming from oxidative phosphorylation, at 95% [4]. This organelle is the interface between physiological and pathophysiological mechanisms. The functions of mitochondria are multiple, including metabolic control and apoptosis, accompanied by the generation of reactive oxygen species (ROS) [4]. Under basal or pathological conditions, the electron transport chain and proton motive force control the electron leakage for ROS production. This is at the origin of a proton gradient and the membrane potential [5]. The controllers of ROS production in mitochondria are numerous, presented in the different complexes, like complexes I and III. In the case of cardiovascular pathologies characterized by a reduced cardiac function, decreased energy production can be observed, inducing cell damage and apoptosis. Considering myocardial ischemia-reperfusion, a mitochondrial respiratory dysfunction of complex I and II lead to an increase of mitochondrial superoxide anion [5].

Furthermore, avoiding cardiac biopsy, a metabolic approach focused on the peripheral blood mononuclear cells (PBMCs) allows improved knowledge of the heart failure pathophysiology [6]. Thus, heart failure relies not only on cardiac defect per se, but it also includes systemic repercussions and particularly reduced PBMCs mitochondrial respiration. The higher the degree of heart failure, the greater the reduction in PBMCs’ mitochondrial oxidative capacities. Such alterations also appeared linked to inflammation [7,8,9].

To date, there is no data concerning PBMCs’ mitochondrial respiration in Htx. Heart transplantation generally normalizes the systolic function of the transplanted heart, but this occurs in an inflammatory context modulated by immunosuppressive therapies. Circulating and local immune cells play a major role in heart transplantation success [10]; we challenged the hypothesis that PBMCs’ mitochondrial respiration might be impaired depending on the patient’s cardiac and/or systemic characteristics.

We, therefore, investigated PBMCs mitochondrial respiratory chain complexes respiration together with superoxide anion, a major reactive oxygen species (ROS), in heart-transplanted patients and matched healthy controls.

## 2. Materials and Methods: Population and Parameters Determined

### 2.1. Population

Twenty Htx aged over 18 years were included during their follow-up and compared to 20 healthy volunteers, matched for sex and age. To avoid cardiac bypass surgery-related systemic inflammation, no patient was included around the perioperative period. The minimum delay since transplantation was one year.

The participants gave their informed consent, and the Ethical Committee of Strasbourg University (CE-2016-91, 15 December 2016) approved the study.

### 2.2. Parameters Determined

Usual clinical, biological and cardiovascular explorations that particularly included echocardiography, coronary artery angiography and right heart catheterization with right ventricular biopsy--using conventional well-controlled methods--were performed in Htx. In addition, 30 mL of venous blood was sampled for PBMCs mitochondrial respiration, and 1 mL was stored on ice for superoxide generation determinations in both Htx and control subjects. The Htx biological characteristics were determined by the hospital laboratory.

### 2.3. Extraction of Circulating Peripheral Blood Mononuclear Cells (PBMCs)

Briefly, blood was gently deposited over a Ficoll density gradient (Eurobio, Lymphocytes separation medium, Courtabeauf France, France) and centrifuged (2100 rpm, 25 min, 18 °C, without brakes). PBMCs were recovered, washed in a DPBS solution (Dulbecco’s Phosphate Buffer Saline 0067M, Hyclone, South Logan, UT, USA) and centrifuged (1600 rpm, 10 min, 18 °C) [11]. If erythrocytes were observed with PBMCs, cells were immersed in a Versalyse-type solution for 20 min allowing the remaining red blood cells to be lysed without affecting the other cell line. Then a new wash with DPBS was carried out. Finally, isolated PBMCs were counted using flow cytometry (Muse Cell Analyser, Merck Millipore, Darmstadt, Germany).

### 2.4. Mitochondrial Respiration of PBMCs

**Figure 1:**
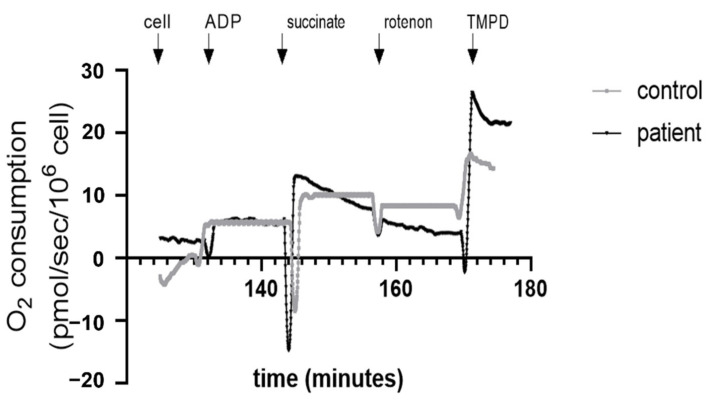
Representative image of mitochondrial respiration of Peripheral Blood Mononuclear Cells (PBMCs) in control and heart transplanted patients.

Oxygen consumption of 2.5 × 10^6^ PBMCs /mL was determined using a high-resolution oxygraph (Oxygraph-2k; Oroboros Instruments, Innsbruck, Austria) at 37 °C with continuous stirring. Cell membranes were permeabilized with saponin (125 µg/mL), and complex I was activated with glutamate (5 mM) and malate (2 mM). This step allows obtaining the basal dioxygen consumption of CI at the leak stage without activation of ATP synthase. Subsequently, different substrates and inhibitors were introduced in the oxygraph’s chamber: (1) ADP (2 mM) induces the activation of ATP synthase (OXPHOS CI) and oxidative phosphorylation via the electron transport coming from complex I. (2) Succinate (25 mM), an activator of the mitochondrial complex II, is added and allows observation of oxidative phosphorylation via the complexes I and II (OXPHOS CI + II). (3) Rotenone (0.5 μM) inhibits complex I, and we observe then oxidative phosphorylation via complex II (OXPHOS CII). (4) TMPD/ascorbate (0.5 mM/0.5 mM) gives electrons directly to the complex IV and activates it (Figure 1). To be more precise, complex IV activity was calculated by the subtraction between step (4) and step (3). All the results are expressed in pmol/s/10^6^ cells.

### 2.5. Measurement of Superoxide Anion

The production of superoxide anion (O_2_^−.^) was determined using electronic paramagnetic resonance (EPR) (E-scan, Bruker-Biospin, Rheinstetten, Germany) at 37 °C [12]. One hour after sampling, blood was mixed with CMH (1-hydroxy-3-methoxycarbonyl-2,2,5,5-tetramethylpytrolidine HI, 200 µM). 40 µL of the mixture obtained was introduced into a glass EPR capillary tube (Noxygen Science Transfer & Diagnostics, Elzach, Germany) and placed inside the cavity of the e-scan spectrometer. A free radical trapper was used to stabilize O_2_^−^. The EPR signal obtained is proportional to the superoxide concentration in the blood sample. The parameters used were: center field g = 3477,452; sweep width 60 G; microwave power 22.21 mW; 2.3 G amplitude modulation; constant time 40.96 ms; conversion time 10.24 ms; number of lag curve points 6. Results are expressed in µmol/min.

### 2.6. Statistical Analysis

Statistical analyses were carried out using GraphPad Prism 8 software (version 3.0), and quantitative data were expressed as mean ± standard error of the mean (SEM). Qualitative variables were described as numbers and percentages. The normality of the value distribution was analyzed according to a Shapiro–Wilk test. Patient and control characteristics were compared according to a non-parametric Mann–Whitney test used to compare the control and the heart transplant groups. The observed correlations were analyzed with a non-parametric Spearman’s test. The *p*-value has been set at 0.05.

## 3. Results

### 3.1. Clinical Characteristics of the Subject

Age and sex were similar in the two groups (Table 1). The delay since transplantation was 9.3 ± 1.8 years and Htx heart rate, systolic and diastolic pressures were respectively 82 ± 3 bpm, 136 ± 4 and 84 ± 3 mm Hg. The main causes leading to transplantation were ischemic cardiomyopathy, followed by dilated and non-obstructive cardiomyopathy. Htx received usual immunosuppressive treatment, including ciclosporine and/or everolimus, cellcept and corticoids. They also received antihypertensive drugs ARBs and/or diuretics, cholesterol-lowering medication, anti-diabetic or other therapy as needed.

### 3.2. Biological Characteristics of the Patients

The main biological characteristics of the Htx are presented in Table 2, as compared to the normal laboratory range. Globally, Htx presented with normal or near normal values considering glycemia, lipids and hepatic function. Creatininemia, as a surrogate of renal function, appeared moderately increased.

**Figure 2:**
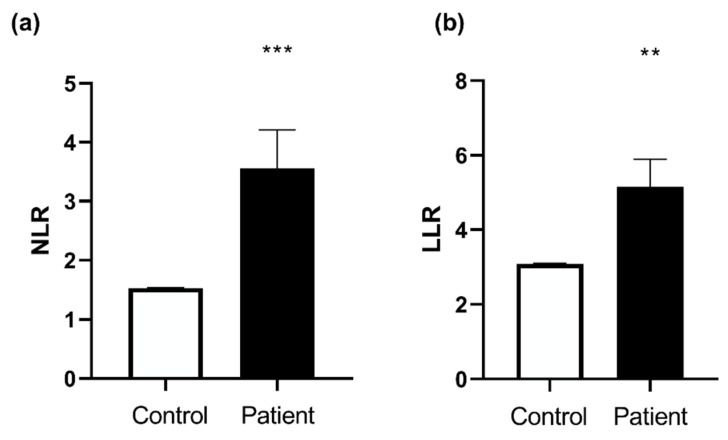
Controls and heart transplant group ratios. (**a**) Neutrophils-to-lymphocyte ratio (NLR), (**b**) Leukocyte-to-lymphocyte ratio (LLR). Values are means ± SEM, ** *p* < 0.01, *** *p* < 0.001. n = 20/group.

Neutrophils-to-lymphocyte ratio (NLR), and leukocyte-to-lymphocyte ratio (LLR), are proposed as markers of inflammation. Neutrophil-to-lymphocyte ratio and platelet-to-lymphocyte ratio as predictors of survival after heart transplantation [13]. They were increased in Htx as compared to the control group (1.53 ± 0.01 vs. 3.56 ± 0.65, respectively, *p* = 0.0002 and 3.09 ± 0.02 vs. 5.16 ± 0.73, respectively, *p* = 0.0025) (Figure 2).

### 3.3. Htx’s Cardiovascular Explorations

#### 3.3.1. Echocardiography

Table 3 shows the main left and right heart echocardiographic characteristics of the Htx. Considering the transplant group, left heart systolic and diastolic functions were normal, with filling pressures within the normal range. Similarly, right ventricular fractional shortening, cardiac index and systolic pulmonary artery pressure remained in the normal range in Htx.

#### 3.3.2. Coronary Artery Angiography

The coronarography was normal in nine patients. Localized irregularities or stenosis of less than 30% were observed in five patients, and significant stenosis was present in the remaining six patients, three of them having either angioplasty or stenting in their history.

#### 3.3.3. Right Heart Biopsy

The systematic right heart biopsy was normal in all but two patients who, in one, demonstrated humoral rejection (pAMR1h) and, in the second, cellular rejection (+ to ++).

### 3.4. Decreased Complex II and Increased Complex IV Mitochondrial Respiration in Htx’s PBMCs

**Figure 3:**
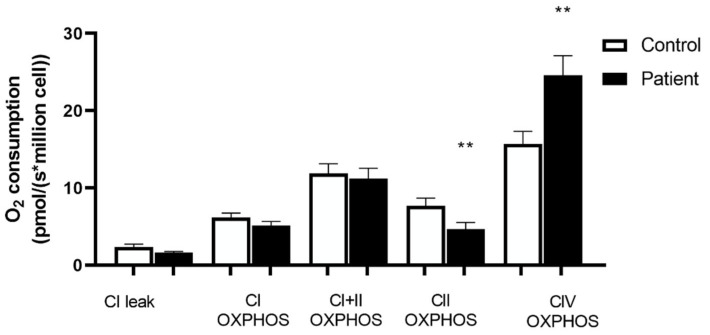
Mitochondrial respiration of Peripheral Blood Mononuclear Cells (PBMCs) in controls and heart transplanted patients. CI: mitochondrial complex I; CI + II: mitochondrial complexes I and II; OXPHOS: mitochondria ADP-activated state of oxidative phosphorylation, CII: mitochondrial complex II. CIV: Mitochondrial complex IV oxidative phosphorylation; Values are means ± SEM, **: *p* < 0.01, n = 20 per group.

Figure 3 shows the mitochondrial respiratory chain activities in controls and Htx. Non-phosphorylating respiration with activation of complex I alone (CI leak) was similar in controls and heart transplant patients (2.37 ± 0.37 vs. 1.61 ± 0.18 pmol/sec/million cell, respectively). Similarly, the O_2_ consumption of oxidative phosphorylation by complex I (CI OXPHOS) shows no significant difference between controls and heart transplant patients (6.16 ± 0.60 vs. 5.12 ± 0.54 pmol/sec/million cell, respectively). Additionally, the O_2_ consumption of oxidative phosphorylation by complex I and II (CI + II OXPHOS) shows no significant difference between controls and heart transplant patients (11.89 ± 1.26 vs. 11.25 ± 1.31 pmol/sec/million cell, respectively). The respiratory control (RCR), calculated as the ratio between oxidation and OXPHOS by complex I, was not significantly different in the two groups.

Nevertheless, the O_2_ consumption of oxidative phosphorylation by complex II (CII OXPHOS) was significantly decreased in heart transplant patients (7.68 ± 0.99 vs. 4.68 ± 0.83 pmol/sec/million cell, respectively; *p* = 0.0067).

On the other hand, CIV OXPHOS: the O_2_ consumption of oxidative phosphorylation by complex IV alone with subtracting CII, is significantly increased in heart transplant patients (15.68 ± 1.67 vs. 24.58 ± 2.56 pmol/sec/million cell, respectively; ** *p* = 0.0035).

### 3.5. Increased Superoxide Anion Production after Heart Transplantation

**Figure 4:**
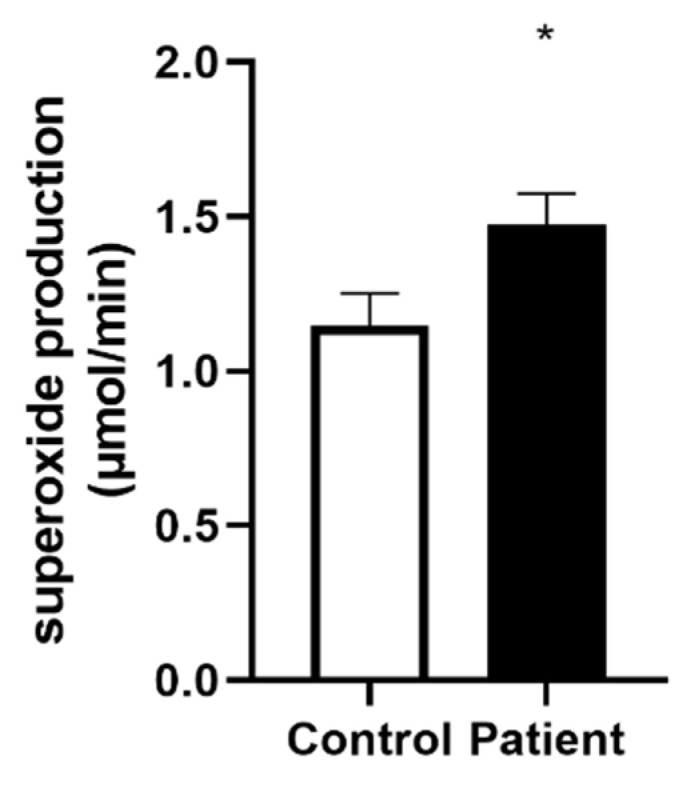
Superoxide anion production in controls and heart transplanted patients. Values are means ± SEM, *: *p* < 0.05, n = 20 in control and 19 in Htx group.

The superoxide anion production was significantly increased in the heart transplant patients’ group (1.15 ± 0.10 vs. 1.47 ± 0.10 µmol/min, for controls vs. Htx patients, *p* = 0.04, Figure 4)**.**

### 3.6. Correlations Related to Complex II Mitochondrial Respiration

**Figure 5:**
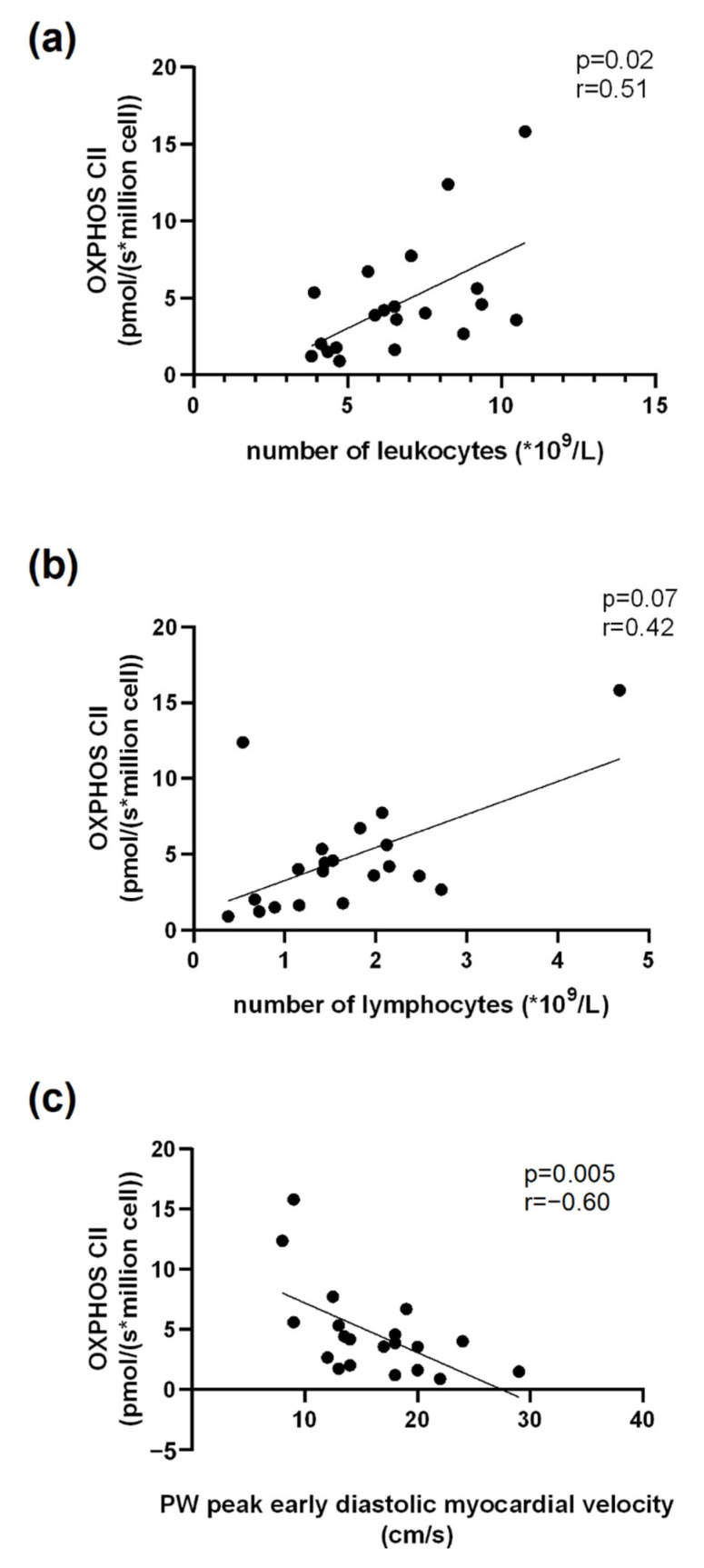
Correlation between OXPHOS CII and number of leukocytes (**a**), number of lymphocytes (**b**) and Posterior wall (PW) peak early diastolic myocardial velocity (cm/s), (**c**).

Concerning blood cell counts, we observed a significant positive correlation between OXPHOS CII and the number of leukocytes (r = 0.51, *p* = 0.02, n = 20, Figure 5a). The correlation between the number of lymphocytes and OXPHOS CII tended to be significant (r = 0.42, *p* = 0.07, n = 20, Figure 5b). Additionally, OXPHOS CII and the posterior wall peak early diastolic myocardial velocity obtained using tissue doppler imaging were negatively correlated (r = −0.60, *p* = 0.05, n = 20, Figure 5c).

### 3.7. Correlations Related to Complex IV Mitochondrial Respiration

**Figure 6:**
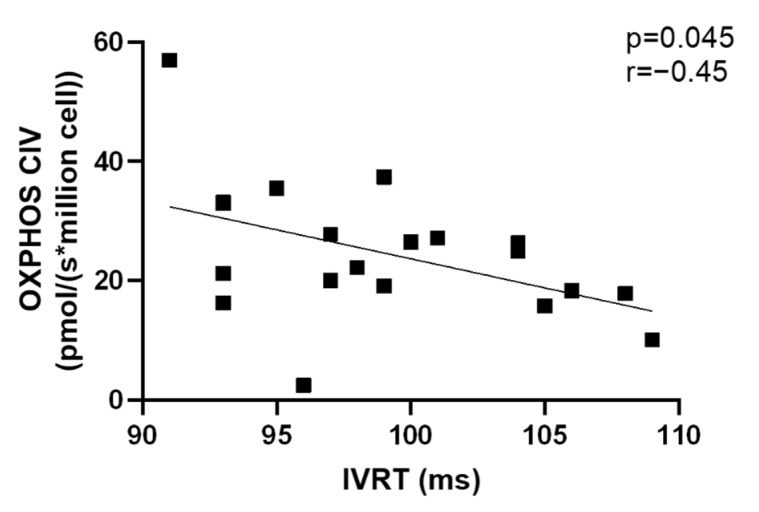
Correlation between OXPHOS CIV and IVRT. IVRT: Isovolumic Relaxation Time.

We observed a significant negative correlation between Htx’s OXPHOS CIV and IVRT (r = −0.45, *p* = 0.04, n = 20). (Figure 6).

**Figure 7:**
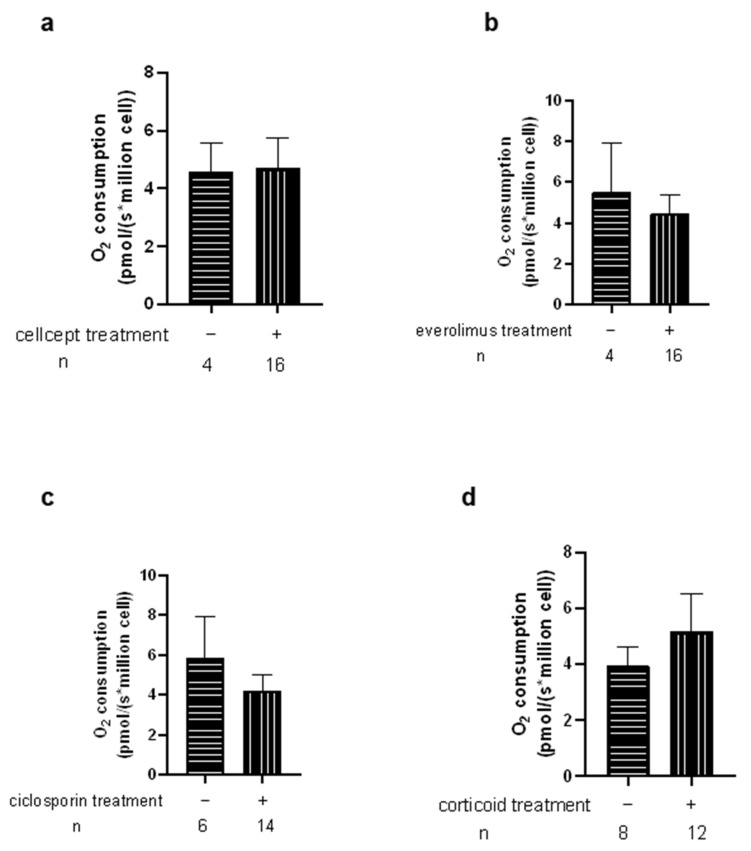
Effects of immunosuppressive therapies on transplant patients PBMCs mitochondrial respiration by complex II (OXPHOS CII). −/+ represents the group without or with specific treatment. n: number of patients in each group. Values are means ± SEM (**a**) Cellcept. (**b**) Everolimus (**c**) Ciclosporin (**d**) Corticoid.

**Figure 8:**
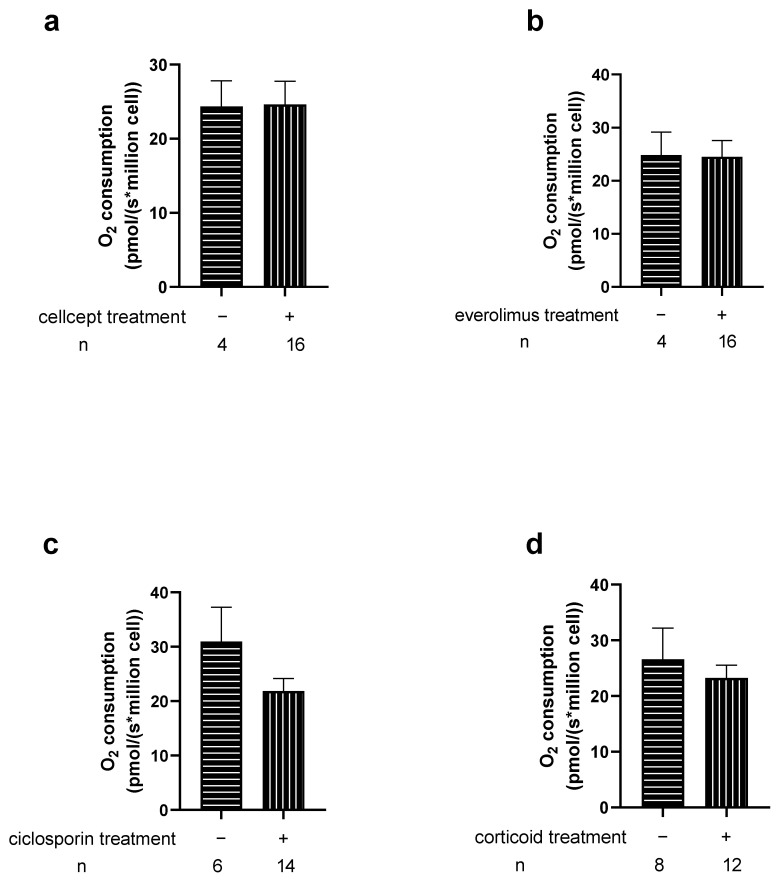
Effects of immunosuppressive therapies on transplant patients PBMCs mitochondrial respiration by complex IV (OXPHOS CIV). −/+ represents the group without or with specific treatment. n: number of patients in each group. Values are means ± SEM. (**a**) Cellcept. (**b**) Everolimus (**c**) Ciclosporin (**d**) Corticoid.

We then investigated the potential relationship between mitochondrial respiration changes in Htx and their immunosuppressive therapies (Figure 7 and Figure 8). We observed no significant difference related to the different therapy used. This was also true when analyzing CMV infection, rejection degree or other parameters.

## 4. Discussion

The main results of this study are that after heart transplantation, PBMC’s mitochondrial respiratory chain complexes show a significant decrease in complex II and an increase in complex IV mitochondrial respiration. This was relatively unexpected in well-being Htx, but subclinical diastolic dysfunction might be involved in these changes. Additionally, lymphopenia and mild inflammation might favor complex II respiration decrease, and acute rejection might be involved in complex IV stimulation.

The mitochondrial respiratory chain is composed of five complexes, and besides calcium handling and participation in apoptosis, its role is to create energy for the cells. This is a major issue, particularly in the heart, an organ needing high oxidative capacity allowing for permanent systolic and diastolic activities. Cardiomyocyte mitochondrial alterations are considered part of heart failure pathophysiology, and authors consistently reported decreases in cardiac mitochondrial complex respiration in several settings, including dilated and ischemic cardiomyopathy [14,15]. However, the need for cardiac biopsy limits this approach, suggesting investigations in a surrogate marker.

In this view, PBMCs mitochondrial respiration appears particularly interesting since it just necessitates blood withdrawal and may reflect cardiac muscle alterations. Studies indicate that PBMCs may function as a feasible non-invasive novel biomarker of heart failure and surrogate for myocardial mitochondrial respiratory function [16,17].

PBMC mitochondrial dysfunction was observed in heart failure patients in relation to inflammation and the severity of the disease [7,9,18]. Interestingly, mitochondrial respiration of cardiomyocytes was reduced by 40 % in acute cellular rejection following heart transplantation [19], further supporting studies on PBMCs mitochondrial respiration in Htx. Indeed, impaired cardiac mitochondrial bioenergetic might be associated with impaired mitochondrial bioenergetic in PBMC [6], thus identifying novel checkpoints in cardiac immune metabolism as potential therapeutic targets in post-transplant care.

To explore the mechanisms involved in the mitochondrial respiration changes observed in our cohort of Htx, we took into account clinical, biological and cardiovascular parameters, including the underlying pathology responsible for heart transplantation, the delay since transplantation, cardiac, coronary, rejection investigations and the different categories of drug given to the patients.

### 4.1. Decreased PBMCs Mitochondrial Respiratory Chain Complex II Respiration after Heart Transplantation

Complex II, called succinate dehydrogenase (SDH), is the sole complex that does not pump protons across the inner mitochondrial membrane and has all of its subunits encoded by nuclear DNA [20,21]. In the electron transport chain, complex II reduces ubiquinone to ubiquinol, and alterations might be related to mutations, which have been observed in cardiomyopathy [22]. Complex II phosphorylating activity decrease has been shown in patients with early-stage HF and might be related to reduced mitochondrial biogenesis or increased mitophagy per mononuclear cell [7].

Complex II deficiency being associated with cancer [23,24] and viral infection [20], we investigated a possible relationship between complex II respiration and the presence of cancer and the viral status in our Htx population. Complex II respiration was not specifically decreased in patients having developed cancer or CMV. Similarly, complex II respiration decline was not associated with increasing age [25], nor with the immunosuppressive regimen, although both ciclosporin and MMF might impair mitochondrial respiration [26,27,28]. On the other hand, ciclosporin can be protective after binding to cyclophilin D, improving thus mitochondrial function and reducing ROS production and inflammation [29]. Further, the potential deleterious effect might have been counterbalanced by mTOR inhibitors that rather improve the mitochondrial function of PBMCs and decrease the level of inflammatory markers [30]. This might also explain the lack of relationship between increased superoxide anion and decreased complex II respiration in our Htx’s PBMC. Incidence of oxidative stress is likely mild in these patients [31], but studies investigating whether the increase in superoxide anion production in Htx might mainly be related to mitochondrial dysfunctions and/or to enzymatic sources like NADPH or xanthine oxidases will be useful.

Interestingly, the study of mitochondrial respiration of endomyocardium in Htx with cardiac allograft vasculopathy showed that maximally coupled respiration of mitochondrial complex I and II was significantly reduced [3]. Our data on PBMCs are in line with these results, albeit we did not find a clear correlation between vasculopathy and complex II respiration in our patients. This might be because many Htx were well-being with conserved left ventricular ejection fraction and no or only a few vasculopathy signs. On the other hand, the significant negative correlation between complex II respirations and the posterior wall peak early diastolic myocardial velocity suggests a role in diastolic function in Htx’s PBMC mitochondrial respiration decrease.

Changes in blood cell count might also likely participate in the complex II alterations observed in Htx. Indeed, as observed in heart failure [6], the increased neutrophil/lymphocyte ratio might have led to decreased PBMC mitochondrial respiration. Such cellular switch with a relative lymphocytopenia related to inflammation and down-regulation of the immune system [32] could lead to a decrease in global PBMC mitochondrial respiration, since neutrophils poorly contribute to the oxygen consumption rate and cellular bioenergetics, as compared to lymphocytes [33]. Accordingly, we observed a correlation between complex II respiration and leucocyte number, and the low number of lymphocytes tended to be associated with a low complex II-related mitochondrial respiration in Htx.

### 4.2. Increased PBMCs Mitochondrial Respiratory Chain Complex IV Respiration after Heart Transplantation

Cytochrome C oxidase (COX), also known as complex IV, is the final enzyme of the electron transport chain system in mitochondria as it is the last electron acceptor [34,35]. This protein is considered an important modulatory location for oxidative phosphorylation (mitochondrial respiration) “OXPHOS” since this is the location where over 90% of oxygen is consumed without the formation of ROS [36,37,38]. In addition, complex IV is known to modulate ROS production and diminish oxidative damage [39].

Thus, increased complex IV activity can be viewed as a compensatory mechanism for the decreased complex II respiration observed in Htx. It might also be related to statin treatment (generally associated with decreased inflammatory markers) since simvastatin increased complex IV mitochondrial respiration in PBMC, as compared to untreated controls, in association with an increase in superoxide production [40].

The inverse correlation observed between isovolumic relaxation time and the activity of complex IV also suggests its implication in diastolic cardiac function, but this will need confirmation, albeit increased complex IV activity has been observed in cardiac ischemia [41].

Interestingly, the two patients presenting with cellular or humoral rejection during the study showed an increase in the activity of complex IV. Although their number is insufficient to conclude definitively, this might be a compensatory activation of the immune response toward an anti-inflammatory effect [42].

Potentially giving coherence to the mitochondrial changes observed in this study, EL Mills et al. proposed that inhibition of succinate oxidation promotes an anti-inflammatory outcome [43].

### 4.3. Limitations of the Study

Although the number of patients included demonstrates changes in PBMC respiration after heart transplantation, a larger population would be useful to determine further the mechanisms involved. Particularly, the hypothesis of a potential link between complex IV stimulation and acute rejection needs investigations likely through a multicenter study in view of the relatively low frequency of acute rejection.

## 5. Conclusions

After successful heart transplantation, PBMCs demonstrated a significant decrease in complex II and an increase in complex IV mitochondrial respirations, together with increased superoxide anion production. Although confirming data observed in the cardiovascular diseases [44], these changes occur in well-being Htx. Subclinical diastolic changes might be involved, and further, complex II respiration alteration likely relates to relative lymphopenia. Complex IV increase might also potentially relate to acute rejection. Both mitochondrial respiration changes might favor anti-inflammatory pathways, and thus, studies are needed to determine whether PBMC’s mitochondrial respiration might be a potential marker of acute rejection and/or mild diastolic dysfunction after heart transplantation.

## Figures and Tables

**Table 1:** Clinical characteristics of the subject.

	Healthy Controls	Heart Transplanted Patients
Gender (M/F)	17/3	18/2
Age (years)	59.6 ± 2.4	59.5± 2.5
Body Mass Index (kg/m^2^)	25.9 ± 0.98	24.4 ± 1.0
**Comorbidity (n, %)**		
Hypertension	0	14, 70%
Diabetes	0	6, 30%
Dyslipidaemia	0	15, 75%
**Initial cardiomyopathy (n, %)**		
Cardiac ischemia	0	7, 35%
Dilated cardiomyopathy	0	3, 15%
Non-obstructive cardiomyopathy	0	3, 15%
Valvular	0	2, 10%
Congenital	0	2, 10%
Rhythmic	0	1, 5%
Toxic	0	1, 5%
Genetic	0	1, 5%

**Table 2:** Biological characteristics of the heart transplanted patients.

	Normal Values (Range)	Htx (Mean ± SEM)
Glycemia (mmol/L)	4.55–6.38	6.28 ± 0.44
Creatinine (umol/L) ± SEM	64–104	137 ± 16
Cholesterol (g/L) ± SEM	1.50–2.00	1.83 ± 0.1 n = 19
LDLc (g/L) ± SEM	<1.60	0.95 ±0.1, n = 19
Triglycerides (g/L) ± SEM	0.35–1.50	1.97 ± 0.24, n = 19
BNP (pg/mL) ± SEM	10–150	221.42 ± 104.42, n = 19
HB (hemoglobin level (g/dL)	12–16	12.81 ± 0.39, n = 20
**Blood Cells Count (×10^9^/L)**		
Leucocytes	4–11	6.72± 0.47, n = 20
Lymphocytes	1–4	1.65 ± 0.21, n = 20
Neutrophils	1.40–7.70	4.29 ± 0.39
**Inflammation**		
CRP (mg/L) ± SEM	<5.0	6.31 ± 1.25, n = 20
NLR (Neutrophil-to-lymphocyte ratio)	1.53 ± 0.01, n = 20	3.56 ± 0.65, n = 20
LLR (Leukocyte-to-lymphocyte ratio)	3.09 ± 0.02, n = 20	5.16 ± 0.73, n = 20

Heart transplanted patients (Htx), LDLc: Low-Density Lipoprotein cholesterol, BNP: brain natriuretic peptide.

**Table 3:** Echocardiographic characteristics of Htx.

	Normal Values	HTx
Left ventricular ejection fraction (%)	>55	63 ± 1.6
Right ventricular fractional shortening (%)	>32	47 ± 1
Cardiac index (L/min/m^2^)	2.5–3.5	3.16 ± 0.11
Systolic pulmonary artery pressures (PAPs) (mmHg)	<35	32.7 ± 1.8
E/A	1–2	1.71 ± 0.09
IVRT (Isovolumic Relaxation Time) (ms)	60–100	99.05 ± 1.19

HTx: Heart transplant patient. E/A: Mitral E and A wave’s ratio.

## Data Availability

The data presented in this study are available on request from the corresponding author.
